# Ecotoxicity of binary mixtures of ILs and inorganic salts of electrochemical interest

**DOI:** 10.1007/s11356-021-17515-1

**Published:** 2021-11-27

**Authors:** Juan José Parajó, Pablo Vallet, Luis Miguel Varela, María Villanueva, Josefa Salgado

**Affiliations:** 1 NAFOMAT Group, Departamentos de Física Aplicada y Física de Partículas, Universidade de Santiago de Compostela, 15782 Santiago de Compostela, Spain; 2 Departamento de Química e Bioquímica, CIQUP – Centro de Investigação em Química da Universidade do Porto, Universidade do Porto, P-4169-007 Porto, Portugal

**Keywords:** Ionic liquids, Ecotoxicity, Smart electrolyte, Microtox^®^

## Abstract

The applicability of ionic liquids (ILs) has increased over the last years, and even new opportunities are becoming a reality, i.e. mixtures of pure IL and inorganic salt as electrolytes for smart electrochemical devices, yet the effects on the environment are almost unknown. In this work, the ecotoxicity of two pure protic ILs (Ethylammonium nitrate and Ethylimidazolium nitrate) and two pure aprotic ILs (butylmethylpyrrolidinium bis(trifluoromethylsulfonyl)imide and butyldimethylimidazolium bis(trifluoromethylsulfonyl)imide) and that of their binary mixtures with inorganic salts with common cation was tested towards changes in the bioluminescence of the bacteria *Alii*v*ibrio fischeri*, using the Microtox® standard toxicity test. EC_50_ of these mixtures was determined over three standard periods of time and compared with the corresponding values to pure ILs. Results indicate that the aprotic ILs are more toxic than protic and that aromatic are more toxic than non-aromatic. The addition of inorganic mono (LiNO_3_), di (Ca(NO_3_)_2_·4H_2_O, Mg(NO_3_)_2_·6H_2_O) and trivalent (Al(NO_3_)_3_·9H_2_O) salts in binary mixtures with EAN was analysed first. The latter was found to induce an important increase in toxicity. Finally, mixtures of IL-inorganic lithium salt (LiNO_3_, for the protic ILs and LiTFSI for the aprotic ILs) toxicity was also studied, which showed toxicity levels strongly dependent on the IL of the mixture.

## Introduction

As is well known, ionic liquids (ILs) are compounds formed entirely by ions which have low melting points. The number of applications seems to be endless since they have still not been fully studied as pure, in mixtures with other compounds or with active pharmaceutical ingredients (APIs) incorporated in their structures besides many other possibilities (Rodríguez and Brennecke 2006; Rana et al. 2010; Silva et al. 2016; Toledo-Hijo et al. 2016; Salgado et al. 2019).

The main characteristic of ILs is the low vapour pressure, which means non-volatility, and therefore, they were, at first, used to substitute traditional industrial solvents, most of which are volatile organic compounds (VOCs), one of the most sources of environmental pollution in the chemical industry (Rogers and Seddon 2003). This led to them being labelled green solvents, although recent studies concluded that the toxicity of some ILs is similar, or even higher, than traditional solvents (Studzinska and Buszewski 2009; Santos et al. 2014).

Aside from non-volatility, other characteristic properties of ILs include high thermal and chemical stability, high viscosity, solubility in water and other solvents, a wide electrochemical window and especially tuneability (Yasuda et al. 2013). All these characteristics make ILs good candidates to be used in high-temperature applications such as lubrication (Otero et al. 2014), desulfurization of fuels (Gutiérrez et al. 2018), batteries (Menne et al. 2013; Balducci 2017; Wang et al. 2020), fuel cells (Nakamoto and Watanabe 2007), fluids in refrigeration systems (Sánchez et al. 2016; Moreno et al. 2018) and APIs (Shamshina and Rogers 2020).

ILs can be divided into two different subclasses depending on their structural characteristics: protic (PILs) and aprotic (AILs) ionic liquids. PILs are formed by the proton transfer from acid to base. Hence, they consist of proton-donor and -acceptor sites, which are responsible for building extended three-dimensional hydrogen bond networks, as in the case of water. AILs are also mainly based on bulky organic cations (*i.e.* pyrrolidinium, imidazolium) with long alkyl chain substituents and on a huge variety of anions (*i.e.* TFSI, FAP, halides). In recent years, significant growth in the structure−property relationships of ILs has been achieved with a better understanding of the intermolecular forces (Salgado et al. 2013; Sánchez et al. 2019).

One of the most cited applications in the literature is electrochemistry. Inorganic salt-doped ionic liquids are highly promising electrolyte media compatible with inorganic and organic electrodes for batteries, supercapacitors and other electrochemical storage devices. Lithium salts are those most used for doping ILs (Menne et al. 2013; Balducci 2017), finding high Li transport numbers and improving ionic conductivity. Moreover, divalent and trivalent salts as well as IL mixtures also showed promising results, improving some properties that further extend the range of application of these compounds (Yang et al. 2020). Salgado et al. (2019) have stated that melting and glass transition temperatures decrease with increasing salt concentration, whilst thermal stability is not significantly affected. Kim et al. (2011) have studied the mixture n-butyl-n-methylpyrrolidinium TFSI with Li TFSI salt, showing a slight decrease in ionic conductivity when salt concentration increases. These papers also comment on the applicability in refrigeration besides the most traditional applications in electrochemistry and other uses.

In addition to good physicochemical properties of these compounds, current European Union environmental legislation, including REACH (Regulation concerning registration, Evaluation, Authorization and Restriction of Chemicals) (Commission 2006), requires the use of safety materials, highlighting the principles of Green Chemistry as prevention, economy, less hazardous chemical synthesis, efficient use of energy, use of renewable raw and biodegradable materials, monitoring of real-time technological processes and provision of an adequate level of chemical safety.

Therefore, it is urgent to establish evaluation procedures to estimate the toxicity of ILs that can readily provide the needed information and reduce costs. *Aliivibrio fischeri* (*A. fischeri*) is a well-known marine luminescent bacterium with a short reproductive cycle, and whose toxicity inference may be extrapolated to a wide variety of aquatic organisms. Thus, it can be effectively applied for toxicological risk assessment (Ventura et al. 2013; Parajó et al. 2019).

It is generally accepted that the structure of ILs has an important influence on the physical and chemical properties as well as in toxicity, although further studies need to be performed. Consequently, the specific choice of cation and anion has an important influence on the ecotoxicity of ionic liquids. It is well known that ILs with aromatic cations are more toxic than non-aromatic ones, mainly due to their water solubility (Ventura et al. 2013). Moreover, imidazolium- and pyridinium-based ILs with a specific anion show the highest harmful effects among other cations, with EC_50_ of 130 mg/L for the 1‐butyl‐3‐methylpyridinium bromide and 5525 mg/L for 1‐butyl‐1‐methylpyrrolidinium bromide as Ibrahim et al. (2017) highlighted in their work. These researchers also underline that the alkyl chain length also has a strong influence on ecotoxicity, as well as in other thermophysical properties such as density and viscosity. As a consequence, EC_50_ is reduced by almost four orders of magnitude, from 3234 mg/L (relatively harmless) for 1‐ethyl‐3‐methylimidazolium chloride to 0.58 mg/L for 1-hexadecyl‐3‐methylimidazolium chloride (highly toxic, according to Passino and Smith classification (Passino and Smith 1987) ). With regard to the anion, in spite of its undoubted influence, the effects depend on the cation moiety; for example, for the cation [Emim]^+^, the EC_50_ are 9213 mg/L and 1631 mg/L for the [Cl]^−^ and [TFSI]^−^ anions, respectively. Nevertheless, for the cation [C_8_mim]^+^, the corresponding values are 2.36 mg/L for chloride and 6.44 mg/L for [TFSI]^−^ (moderately toxic), which shows that the trend followed for the anions can change with the alkyl chain length.

Furthermore, several prediction models have been used for estimating the toxicity of ILs towards different endpoints, most of them through the QSAR (quantitative structure–activity relationship) technique (Luis et al. 2010; Ibrahim et al. 2017). A recent paper (Kang et al. 2020) developed an interesting method for predicting the toxicity of ILs from the electrostatic potential surface area of separate cation and anion of 142 ILs using a learning machine algorithm, obtaining excellent results. Nevertheless, in the most recent literature, toxicity predictive methods are gaining ground on experimental methods, which are time-consuming and more expensive. There is still a significant gap in the knowledge of the harmful effects of ILs, both pure and in mixtures with other solvents or salts.

With the aim of contributing to the enlargement of the database of toxic effects of ILs and the consequent improvement of the knowledge of the relationship between toxicity and structure, the ecotoxicity of two protic ILs (ethylammonium nitrate (EAN) and ethylimidazolium nitrate (EIm NO_3_)) and two aprotic ILs (butylmethylpyrrolidinium bis(trifluoromethylsulfonyl)imide (C_4_C_1_pyrr TFSI) and butyldimethylimidazolium bis(trifluoromethylsulfonyl)imide (C_4_C_1_C_1_Im TFSI)) was tested towards changes on the bioluminescence of the bacteria *A. fischeri*, using the Microtox® standard toxicity test. Additionally, changes in the ecotoxicity as a consequence of the doping of these ILs with different salts with electrochemical interest were also determined. Firstly, the doping of the corresponding lithium salt (LiNO_3_, for the protic ILs and LiTFSI for the aprotic IL) was evaluated, and finally, effects of mono (LiNO_3_), di (Ca(NO_3_)_2_·4H_2_O, Mg(NO_3_)_2_·6H_2_O) and trivalent (Al(NO_3_)_3_·9H_2_O) salts were also estimated for EAN. The effective concentration (EC_50_) of these mixtures was determined over three standard periods of time, namely 5 min, 15 min and 30 min, and compared with the corresponding values to pure ILs.

## Materials and methods

### Chemicals

The main characteristics of the four selected ILs (EAN, EIm NO_3_, C_4_C_1_pyrr TFSI and C_4_C_1_C_1_Im TFSI) and the inorganic salt (LiNO_3_, Ca(NO_3_)_2_·4H_2_O, Mg(NO_3_)_2_·6H_2_O, Al(NO_3_)_3_·9H_2_O and LiTFSI) are shown in Table 1. Ionic liquids were dried in a high vacuum under constant stirring for at least 24 h, and the water content, measured by Karl Fischer titration, was below 100 ppm for all liquids. Calcium, magnesium and aluminium nitrate are hydrated salts with four, six and nine water molecules, respectively, and their purity was analysed by EDTA titration. Saturated mixtures of these ILs with lithium salt with common anion were prepared, and salt concentration, in molality and molar fraction, and mixture molecular mass are shown in Table 2. Similarly, saturated mixtures of EAN with lithium, calcium, magnesium and aluminium nitrate salts were also prepared. Saturated concentrations and the molecular mass of the mixtures are summarised in Table 3. All these mixtures were obtained by mixing components, ionic liquid and salt as supplied, with the help of an ultrasound bath and a magnetic stirrer for at least 48 h. The saturated concentrations were determined by increasing molality in intervals of 0.5 mol/kg until precipitation is observed at room temperature (Salgado et al. 2019).

**Table 1:**
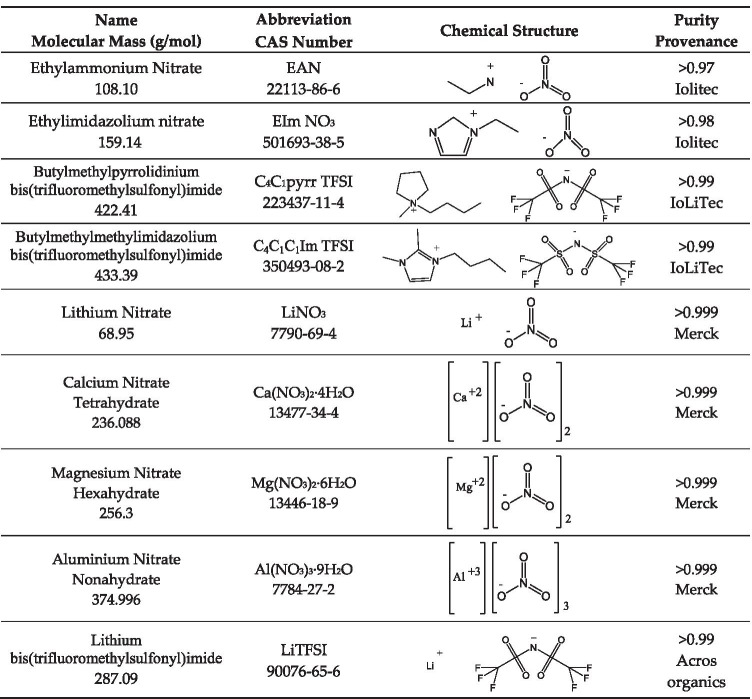
Chemical structure, identification number, molecular mass and purity of ILs and salts

**Table 2:** Lithium salt saturation concentration (molality and molar fraction) in mixtures IL+salt and molecular mass (M_m_) for the mixtures

*Mixture*	EAN +LiNO_3_	C_4_C_1_pyrr TFSI + LiTFSI	EIm NO_3_ + LiNO_3_	C_4_C_1_C_1_ImTFSI + LiTFSI
Molality (mol/l)	2.000	1.500	2.000	1.000
*x*_salt_	0.178	0.388	0.241	0.399
M_m_ (g/mol)	123.01	604.31	181.08	204.83

**Table 3:** Salt saturation concentration (molality and molar fraction) in mixtures EAN + salt and molecular mass (M_m_) for the mixtures

*Mixture*	EAN +LiNO_3_	EAN + Ca(NO_3_)_2_·4H_2_O	EAN + Mg(NO_3_)_2_·6H_2_O	EAN + Al(NO_3_)_3_·9H_2_O
Molality (mol/l)	2.000	1.000	2.000	2.000
*x*_salt_	0.178	0.270	0.446	0.540
M_m_ (g/mol)	123.01	133.62	163.51	189.17

#### Experimental section

Acute toxicity was assessed by determining the luminescence inhibition of the rod‐shaped Gram‐negative marine bacteria *Aliivibrio fischeri* (*A. fischeri*), that bioluminesces through a population‐dependent mechanism called quorum sensing sensitive to a wide variety of toxic substances (Ibrahim et al. 2017). Standard Microtox® liquid phase assays (M500 Analyser – modern water) was used for this proposal. This method is currently one of the most widespread and well-known toxicological bioassays due to its simplicity and quick measurements (Johnson 2005; Chang et al. 2013; Ventura et al. 2014; Parajó et al. 2019). After exposing the bacteria at 15℃ to each different IL or IL+salt aqueous solution (from 0 to 81.9%), the light output at 5 min, 15 min and 30 min was measured and compared with a blank control sample. The concentration of the sample (mg/L) which produces a 50%, 20% and 10% luminescence inhibition after exposure at the three selected times (5 min, 15 min and 30 min) is designated as effective concentration (EC_50_, EC_20_ and EC_10_, respectively) and is calculated, together with the corresponding 95% confidence intervals, through nonlinear regression, using the least-squares method to fit the data to the logistic equation (Parajó et al. 2019). The drop in bioluminescence whilst increasing the concentration of the sample constitutes an integrated measure of the physiological impairment of the bacteria, hence demonstrating the toxic effect of the studied compound (Ventura et al. 2014).

With the aim to clarify the methodology, Table 4 indicates the initial concentrations (mg/L) of the mixture stock solutions prepared for the Microtox® measurements.

**Table 4:** Initial concentrations (mg/L) of pure ILs and binary mixture stock solutions prepared for the Microtox® measurements

Compound	Concentration/mg·L^−1^
EAN	44.461 · 10^3^
EAN + LiNO_3_ 2m	50.850 · 10^3^
EAN + Ca(NO_3_)_2_ 1m	43.346 · 10^3^
EAN + Mg(NO_3_)_2_ 2m	36.893 · 10^3^
EAN + Al(NO_3_)_3_ 2m	3.340 · 10^3^
C_4_C_1_pyrr TFSI	5.394 · 10^3^
C_4_C_1_pyrr TFSI + LiTFSI 1.5m	5.929 · 10^3^
EIm NO_3_	61.172 · 10^3^
EIm NO_3_ + LiNO_3_ 2m	15.915 · 10^3^
C_4_C_1_C_1_Im TFSI	14.590 · 10^3^
C_4_C_1_C_1_Im TFSI + LiTFSI 1m	2.689 · 10^3^

In this work, two classifications were used to distinguish the toxicity of the compounds. The first one is the widely used and proposed by Passino and Smith (1987) based on the values of EC_50_ at 30 min: EC_50_ > 1000 mg/L, relatively harmless; 100 mg/L < EC_50_ < 1000 mg/L, practically harmless; 1 mg/L < EC_50_ < 100 mg/L, toxic; 0.1 mg/L < EC_50_ < 1 mg/L, highly toxic; and 0.01 mg/L < EC_50_ < 0.1 mg/L, extremely toxic.

The other classification is based on the studies of Chang et al. (2013), who used the concept of toxicity units, calculated by

$$TU=\frac{100}{{EC}_{50}}$$

EC_50_ (in mg/L) was measured after 15 min of exposition. Thus, the toxicity steps are defined as follows: TU < 1, non-toxic; 1 < TU < 10, toxic; 10 < TU < 100, very toxic; and TU > 100, extremely toxic.

##### Results and discussion

### Toxicity of pure ILs

A set of ecotoxicity parameters obtained (EC_10_, EC_20_ and EC_50_) for four ILs, two protic and two aprotic, towards the toxicity endpoint of bioluminescence of the bacteria *A. fischeri* is reported in this work. Although the most used parameter is EC_50_, which is the concentration for a 50% of reduction in the luminescence of the bacteria, EC_10_ and EC_20_ (concentrations to reduce 10% and 20% regarding the initial luminescence, respectively) also provide intermediate toxicity references. Hence, a more complete ecotoxicological characterisation of these compounds is included. Furthermore, EC_10_ and EC_20_ are starting points for the estimation of the lowest observed effect concentration. EC_10_ is particularly useful as a reliable parameter of the effects independent of concentration or for the lowest environmental risk compounds (Ventura et al. 2014). Tables 5, 6 and 7 present the values of EC_10_, EC_20_ and EC_50_, respectively, of pure ILs for 5 min, 15 min and 30 min of exposition.

**Table 5:** EC_10_ effective concentration values in mg/L and the respective 95% confidence intervals, obtained after 5 min, 15 min and 30 min of exposure of the marine bacteria *A. fischeri*

IL/mixture	EC_10_ 5 min / mg·L^-1^	EC_10_ 15 min / mg·L^-1^	EC_10_ 30 min / mg·L^-1^
	(Lower; upper) limits	(Lower; upper) limits	(Lower; upper) limits
**Pure ionic liquids**			
EAN	2304.89 (248.43; 4361.05)	1609.79 (560.06; 3163.56)	1517.65 (332.07; 2703.22)
EIm NO_3_	100.10 (21.33; 179.99)	103.80 (22.16; 184.19)	127.39 (37.59; 214.25)
C_4_C_1_pyrr TFSI	438.08 (225.18; 650.98)	254.32 (146.51; 362.18)	170.23 (93.44; 247.12))
C_4_C_1_C_1_Im TFSI	23.25 (0.00; 50.07)	20.96 (7.96; 33.95)	20.34 (12.63; 28.05)
**IL + inorganic salt-saturated mixtures**			
EAN + LiNO_3_ 2m	6842.44 5316.88; 8368.00)	5920.72 (3841.85; 7999.89)	4701.17 (1744.45; 7658.88)
EAN +Ca(NO_3_)_2_·4H_2_O 1m	2732.16 (1304.74; 4159.58)	1938.08 (632.90; 3046.27)	1059.24 (263.33; 1849.14)
EAN +Mg(NO_3_)_2_·6H_2_O 2m	5469.35 (3551.43; 7387.26)	7049.21 (5925.65; 8174.77)	8409.28 (6357.53; 10461.04)
EAN + Al(NO_3_)_3_·9H_2_O 2m	8.04 (3.01; 13.06)	17.96 (12.40; 21.53)	14.30 (10.29; 18.31)
EIm NO_3_ +LiNO_3_ 2m	232.85 (9.66; 455.18)	251.51 (6.98; 496.73)	263.96 (10.87; 515.83)
C_4_C_1_pyrr TFSI + LiTFSI 1.5m	35.88 (17.01; 51.49)	23.22 (6.87; 39.12)	21.12 (8.52 ;35.82)
C_4_C_1_C_1_Im TFSI + LiTFSI 1m	6.35 (0.15; 12.56)	5.26 (2.46; 8.07)	5.45 (3.64; 7.26)

**Table 6:** EC_20_ effective concentration values in mg/L and the respective 95% confidence intervals, obtained after 5 min, 15 min and 30 min of exposure of the marine bacteria *A. fischeri*

IL/mixture	EC_20_ 5 min / mg·L^-1^	EC_20_ 15 min / mg·L^-1^	EC_20_ 30 min / mg·L^-1^
	(Lower; upper) limits	(Lower; upper) limits	(Lower; upper) limits
**Pure ionic liquids**			
EAN	4314.31 (1548.95; 7081.66)	3236.68 (951.77; 5522.60)	3012.33 (1264.99; 4761.67)
EIm NO_3_	195.44 (79.12; 312.90)	194.19 (79.98; 310.53)	223.45 (105.10; 342.82)
C_4_C_1_pyrr TFSI	684.04 (441.90; 926.09)	416.73 (286.18; 545.93)	289.18 (192.91; 386.85)
C_4_C_1_C_1_Im TFSI	46.34 (5.74; 86.95)	39.09 (20.78; 57.40)	36.45 (26.05; 46.85)
**IL + inorganic salt-saturated mixtures**			
EAN + LiNO_3_ 2m	8892.60 (7412.30; 10373.90)	7495.94 (5603.85; 9386.04)	6145.81 (3301.99; 8988.58)
EAN +Ca(NO_3_)_2_·4H_2_O 1m	3939.68 (2427.26; 5450.11)	2858.82 (1475.52; 4240.12)	1804.56 (800.29; 2808.82)
EAN +Mg(NO_3_)_2_·6H_2_O 2m	7471.07 (5520.95; 9421.19)	8933.00 (7887.15; 9979.85)	10222.13 (8450.60; 11994.66)
EAN + Al(NO_3_)_3_·9H_2_O 2m	15.21 (8.47; 22.96)	23.30 (18.29; 27.31)	19.25 (15.84; 23.65)
EIm NO_3_ +LiNO_3_ 2m	423.67 (119.10; 727.77)	435.29 (118.19; 753.03)	442.20 (125.48; 759.18)
C_4_C_1_pyrr TFSI + LiTFSI 1.5m	89.11 (56.09; 123.44)	51.51 (23.58; 79.99)	44.11 (23.08; 64.29)
C_4_C_1_C_1_Im TFSI + LiTFSI 1m	13.01 (3.31; 22.72)	10.09 (6.00; 14.18)	9.88 (7.39; 12.37)

**Table 7:** EC_50_ effective concentration values in mg/L and the respective 95% confidence intervals, obtained after 5 min, 15 min and 30 min of exposure of the marine bacteria *A. fischeri*

IL/mixture	EC_50_ 5 min / mg·L^-1^	EC_50_ 15 min / mg·L^-1^	EC_50_ 30 min / mg·L^-1^
	(Lower; upper) limits	(Lower; upper) limits	(Lower; upper) limits
**Pure ionic liquids**			
EAN	12582.07 (8186.64; 16977.50)	10665.47 (6650.14; 14680.80)	9711.63 (6561.46; 12860.79)
EIm NO_3_	612.55 (395.90; 828.01)	573.77 (372.29; 774.55)	597.89 (408.00; 785.08)
C_4_C_1_pyrr TFSI	1463.91 (1162.13; 1765.69)	964.58 (791.32; 1137.88)	714.43 (577.92; 851.21)
C_4_C_1_C_1_Im TFSI	150.44 (72.43; 228.49)	113.32 (82.29; 144.35)	98.70 (82.39; 115.01)
**IL + inorganic salt-saturated mixtures**			
EAN + LiNO_3_ 2m	13911.23 (12469.75; 15232.70)	11210.37 (9613.17; 12808.57)	9706.72 (7233.87; 12179.58)
EAN +Ca(NO_3_)_2_·4H_2_O 1m	7354.41 (5672.26; 9036.56)	6064.77 (4343.18; 7784.36)	4502.32 ( 3067.63; 5937.02)
EAN +Mg(NO_3_)_2_·6H_2_O 2m	12724.84 (10834.31; 14615.38)	13384.92 (12522.40; 14247.45)	14266.92 (13128.37; 15403.46)
EAN + Al(NO_3_)_3_·9H_2_O 2m	45.22 (32.45; 58.98)	37.34 (33.10; 41.59)	32.25 (29.84; 36.65)
EIm NO_3_ +LiNO_3_ 2m	1178.11 (691.19; 1665.78)	1114.08 (644.19; 1583.21)	1073.03 (626.44; 1520.48)
C_4_C_1_pyrr TFSI + LiTFSI 1.5m	453.19 (360.94; 547.22)	208.10 (142.51; 274.71)	149.60 (108.86; 189.91)
C_4_C_1_C_1_Im TFSI + LiTFSI 1m	44.26 (24.69; 63.82)	30.63 (23.26; 40.00)	27.24 (23.17; 31.32)

**Fig. 1:**
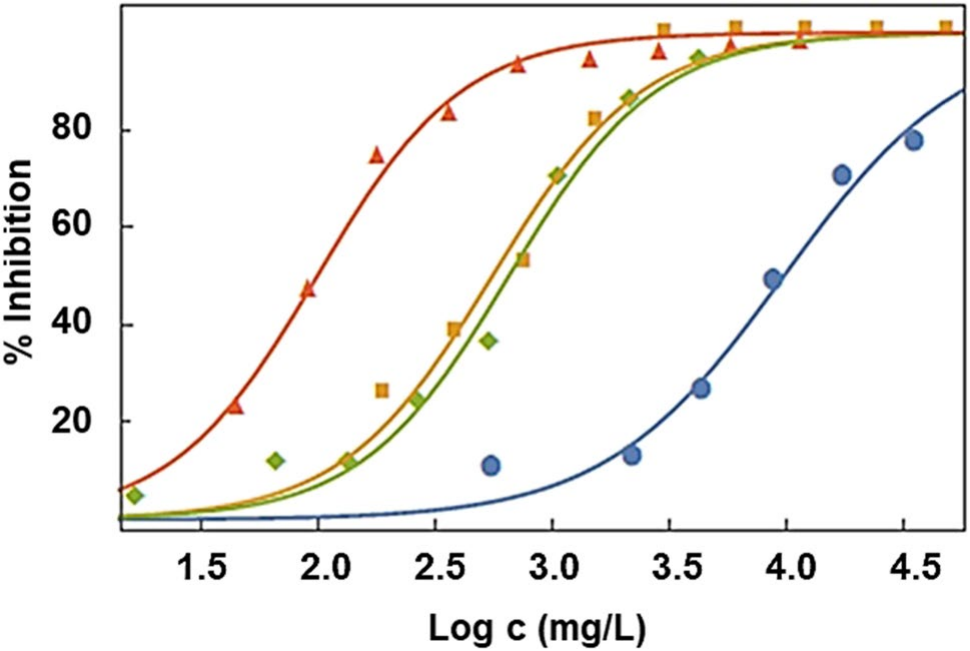
Inhibition of bioluminescence for 30 min of exposure against the logarithm of the concentration of the four pure ionic liquids: (red triangle) C_4_C_1_C_1_Im TFSI, (green rhombus) C_4_C_1_pyrr TFSI, (orange square) EIm NO_3_ and (blue circle) EAN. Lines represent the fitting of the experimental results to Eq. (1)

Figure 1 shows the behaviour of inhibition of the bioluminescence of *Aliivibrio fischeri* bacteria versus the concentration of the four pure ionic liquids. As expected, inhibition increases with the concentration, according to Eq. (1).

1 $$I\left(\%\right)=\frac{100}{1+{10}^{B(A-logc)}}$$

where *I* is the percentage of inhibition, *c* is the concentration, and *A* and *B* are adjusted parameters, whose values for the pure ILs and the corresponding absolute average percentual deviation, AAD (Eq. (2)), are shown in Table 8.

**Table 8:** Adjustable parameters (with the standard deviation) of the logistic equation (Eq. (1)) and the absolute average percentual deviation (AAD) (Eq. (2))

Sample	A	B	AAD (%)
EAN	3.99 ± 0.04	1.14 ± 0.13	18.1
EIm NO_3_	2.75 ± 0.04	1.35 ± 0.17	6.3
C_4_C_1_pyrr TFSI	2.81 ± 0.04	1.38 ± 0.15	24.6
C_4_C_1_C_1_Im TFSI	2.00 ± 0.02	1.40 ± 0.10	3.2
EAN + LiNO_3_ 2m	3.96 ± 0.03	2.6 ± 0.4	52.3
EAN +Ca(NO_3_)_2_·4H_2_O 1m	3.67 ± 0.06	1.5 ± 0.3	13.7
EAN +Mg(NO_3_)_2_·6H_2_O 2m	4.12 ± 0.01	3.4 ± 0.2	41.4
EAN + Al(NO_3_)_3_·9H_2_O 2m	1.49 ± 0.01	2.47 ± 0.10	1.7
EIm NO_3_ +LiNO_3_ 2m	2.16 ± 0.04	1.35 ± 0.08	6.3
C_4_C_1_pyrr TFSI + LiTFSI 1.5m	2.20 ± 0.04	1.18 ± 0.10	11.4
C_4_C_1_C_1_Im TFSI + LiTFSI 1m	1.45 ± 0.02	1.39 ± 0.08	4.5

2 $$AAD=\frac{100}{N}\sum \left|\frac{{I}_{exp}-{I}_{adj}}{{I}_{exp}}\right|$$

where *I*_exp_ is the experimental percentage of inhibition, *I*_adj_ is the inhibition obtained from the adjustment, and *N* is the number of experimental data.

It is widely acknowledged that the bacterial bioluminescence reactions are indicative of the cellular metabolism of the bacteria, and a reduction in bioluminescence implies a decrease in cellular respiration (Perales et al. 2016). Consequently, the trend followed by these four ILs is C_4_C_1_C_1_Im TFSI > C_4_C_1_pyrr TFSI ≈ EIm NO_3_ > EAN. A similar tendency was obtained for EC_20_ 30 min, but for the values EC_50_ at 30 min, the trend obtained is slightly different: C_4_C_1_C_1_Im TFSI > EIm NO_3_ > C_4_C_1_pyrr TFSI > EAN. These results agree with the previous idea that the protic and non-aromatic ILs are less toxic than aprotic and aromatic ones.

**Fig. 2:**
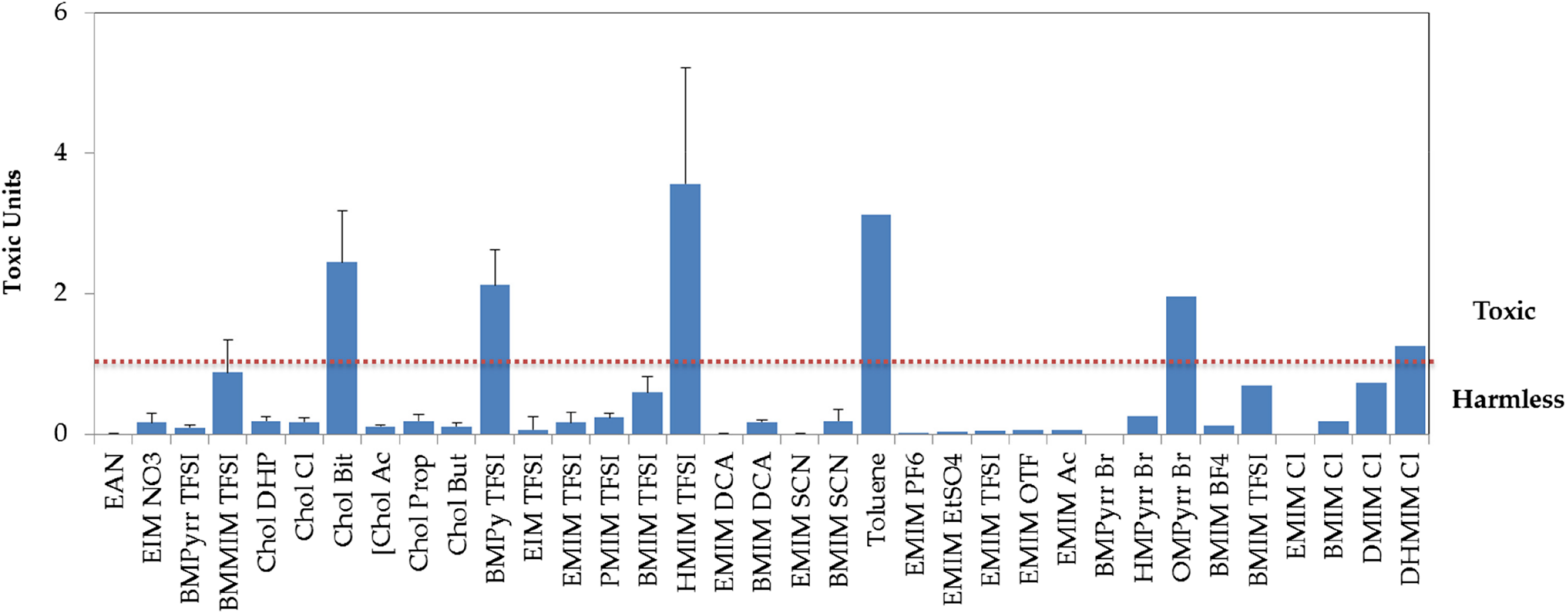
Comparison of the toxicity level (Chang et al. 2013) of the ILs studied here and some of the literature (error bars were included if the uncertainty intervals were published). Cholinium dihidrogen phosphate (Chol DHP), choline chloride (Chol Cl), cholinium bitartatre (Chol Bit), choline acetate (Chol Ac), choline propanoate (Chol Prop) and choline butanoate (Chol But) (Ventura et al. 2014); 1-butyl-4-methylpyridiniumbis (trifluoromethylsulfonyl)imide (BMPy TFSI), 1-ethylimidazolium bis(trifluoromethylsulfonyl) imide (EMIM TFSI), 1‐ethyl‐3‐methylimidazolium bis(trifluoromethylsulfonyl) imide (EMIM TFSI), 1‐propyl‐3‐methylimidazolium bis(trifluoromethylsulfonyl) imide (PMIM TFSI), 1‐butyl‐3‐methylimidazolium bis(trifluoromethylsulfonyl) imide (BMIM TFSI), 1‐ethyl‐3‐methylimidazolium bis(trifluoromethylsulfonyl) imide (EMIM TFSI), 1‐hexyl‐3‐methylimidazolium bis(trifluoromethylsulfonyl) imide (HMIM TFSI), 1‐ethyl‐3‐methylimidazolium dicyanamide (EMIM DCA), 1‐butyl‐3‐methylimidazolium dicyanamide (BMIM DCA), 1‐ethyl‐3‐methylimidazolium thiocyanate (EMIM SCN), 1‐butyl‐3‐methylimidazolium thiocyanate (BMIM SCN) (Delgado-Mellado et al. 2019); toluene (Hernández-Fernández et al. 2015); 1‐ethyl‐3‐methylimidazolium chloride (EMIM Cl), 1‐ethyl‐3‐methylimidazolium hexafluorophosphate (EMIM PF_6_), 1‐ethyl‐3‐methylimidazolium ethylsulphate (EMIM EtSO_4_), 1‐ethyl‐3‐methylimidazolium bis((trifluoromethyl) sulfonyl)amide (EMIM TFSI), 1‐ethyl‐3‐methylimidazolium triflate (EMIM OTF), 1‐ethyl‐3‐methylimidazolium acetate (EMIM Ac), 1‐butyl‐1‐methylpyrrolidinium bromide (BMPyrr Br), 1‐hexyl‐1‐methylpyrrolidinium bromide (HMPyrr Br), 1‐octyl‐1‐methylpyrrolidinium bromide (OMPyrr Br), 1‐butyl‐3‐methylimidazolium tetrafluoroborate (BMIM BF_4_), 1‐butyl‐3‐methylimidazolium chloride (BMIM Cl), 1‐butyl‐3‐methylimidazolium bis((trifluoromethyl) sulfonyl)amide (BMIM TFSI) (Ibrahim et al. 2017); 1-decyl-3-methylimidazolium chloride (DMIM Cl), 1-hexadecyl-3-methylimidazolium chloride (DHMIM Cl) (Kang et al. 2020)

Taking into account the two criteria set out in the previous section, EAN, with EC_50_ at 30 min of 12582 mg/L value, higher than 1000 mg/L and TU≈9.10^−3^, is placed in the lowest group of toxicity and is harmless. The harmfulness of TFSI-based aprotic ILs depends on the cation, being practically harmless (714 mg/L and TU = 0.1) for the C_4_C_1_pyrr TFSI, and for the C_4_C_1_C_1_Im TFSI (almost) toxic according to both criteria (1 mg/L < 98.70 mg/L < 100 mg/L and TU≈1). A comparison of toxicity of 32 ILs between the most used as well as toluene (one of the most used VOCs) is shown in Fig. 2. The majority of these ILs are essentially harmless, and imidazolium cations with long alkyl chain length and TFSI^−^ anion seem to be the most toxic, even more than toluene.

Microtox toxicity screening has been widely applied to different ionic liquids families; nevertheless, results of the selected ILs of this work are scarce. The case of EAN is especially striking, which became the first synthetized IL more than a century ago. Ventura et al. (2013) obtained values of EC_50_ at 5 min and 15 min of 130.85 mg/L and 87.23 mg/L, respectively, for C_4_C_1_C_1_Im TFSI. These values are in very good concordance with our results for this IL and with that of Delgado-Mellado et al. (2019), who found values of 141 mg/L and 100 mg/L for the EC_50_ at 5 min and 15 min, respectively. Montalbán et al. (2016) have also found lower values for EC_50_ at 30 min for pure EAN, being relatively harmless and non-toxic according to both classifications indicated in this work. In the case of C_4_C_1_pyrr TFSI, Viboud et al. (2012) have found EC_50_ at 15 min of 219 mg/L, which is lower than our value but corresponds to the same toxicity group. Additionally, Ventura et al. (2013) analysed the effect of some TFSI-based ILs with the same alkyl length, being pyrrolidinium one of the most harmless of these aprotic ILs, also in agreement with our observations.

As a general conclusion, the results reported here confirm the idea that the protic ILs are generally less toxic than aprotic ones, and the non-aromatic ones are less toxic than aromatic ones. Additionally, the role of water solubility is important, where lower toxicity is related to higher hydrophilicity in every group (Peric et al. 2013; Ventura et al. 2013).

### Toxicity of salts –EAN mixtures

**Fig. 3:**
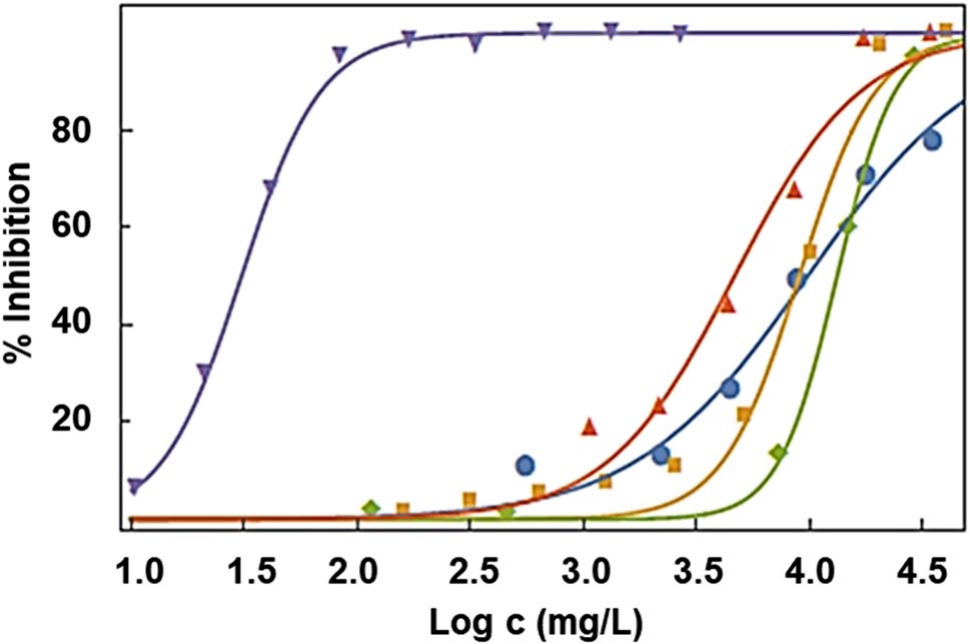
Inhibition of bioluminescence for 30 min of exposure against the logarithm of the concentration of the (blue circle) pure EAN, and mixtures with the four nitrate salts: (orange square) EAN-lithium nitrate mixture, (green rhombus) EAN-magnesium nitrate mixture, (up red triangle) EAN-calcium nitrate mixture and (down blue triangle) EAN-aluminium nitrate mixture. Lines represent the fitting of the experimental results to Eq. (1)

The second part of this work studies the effect on toxicity regarding *A. Fischeri* bioluminescence inhibition of different salt additions to EAN IL. Figure 3 presents the percentage of bioluminescence inhibition of the bacteria with regards to the concentration of EAN – nitrate salt-saturated solutions. All of them follow a logistic equation (1), and the adjusted parameters values and AAD are collected in Table 8. Additionally, the values of EC_10_, EC_20_ and EC_50_ of these samples for 5 min, 15 min and 30 min of exposition are also shown in Tables 5, 6 and 7. Results showed that mixtures of EAN with the mono and divalent salts did not significantly affect the toxicity of the mixture with regards to the pure ionic liquid, but the addition of the aluminium salt had an important effect on the bioluminescence of *A. Fischeri*. This mixture is toxic in both classifications used in this article, as can be seen in Table 9. This behaviour may be related to the well-known antimicrobial and antibacterial effects of aluminium salts which are also related to the acidification of the sample as a consequence of this salt addition (being the pH lower than 4 for this salt, whereas for the other salt, it is always higher than 5) (Guida et al. 1991). As is widely acknowledged, aluminium in a solid state plays a key role in the environment, but due to high reactivity, it is difficult to find in a free state in nature. At neutral or weakly acidic pH, it is present in the form of insoluble oxides and aluminosilicates. However, for high acidic media, aluminium is solubilised into a phytotoxic form (Matsumoto 2000). Since under these acidic conditions, it is a polyvalent cation that binds strongly to negative charges, usually the carboxyl groups in cell wall molecules affect cell division, cell extension or nutrient transport and provoke cell growth disruption and serious perturbation of the normal metabolism of living organisms (Haug 1984; Jones and Ryan 2016). Contrarily, Li^+^, Ca^+2^ and Mg^+2^ cations do not exert any appreciable influence on the toxicity of the IL. Indeed, these cations play different beneficial functions in cells since they are oligoelements, i.e. metal or metalloid ions that have important roles such as constituents of cell tissues or as catalysers of chemical reactions of the cell metabolism (Carvalho et al. 2015). Specifically, lithium plays a special role in brain diseases, for example, through the inhibition of the inositol, which is sugar involved in the ability of neurons to exchange signals. Lithium lowers inositol levels, calming overactive neurons (Pilcher 2003). Mg^+2^ and Ca^+2^ have intermediate binding strengths to organic ligands. In the particular case of Ca^+2^, it works for a charge carrier and for signal transmission inside the cells (Crichton 2017).

**Table 9:** Toxicity identification of the EAN-nitrate salt mixtures using the criteria of Passino and Smith (1987) and Chang et al. (2013)

IL/mixture	Passino and Smith (1987)	Chang et al. (2013)
EAN	Relatively harmless	Non-toxic
EAN + LiNO_3_ 2m	Relatively harmless	Non-toxic
EAN + Ca(NO_3_)_2_ 1m	Relatively harmless	Non-toxic
EAN + Mg(NO_3_)_2_ 2m	Relatively harmless	Non-toxic
EAN + Al(NO_3_)_3_ 2m	Toxic	Toxic

### Toxicity of IL-lithium salt mixtures

**Fig. 4:**
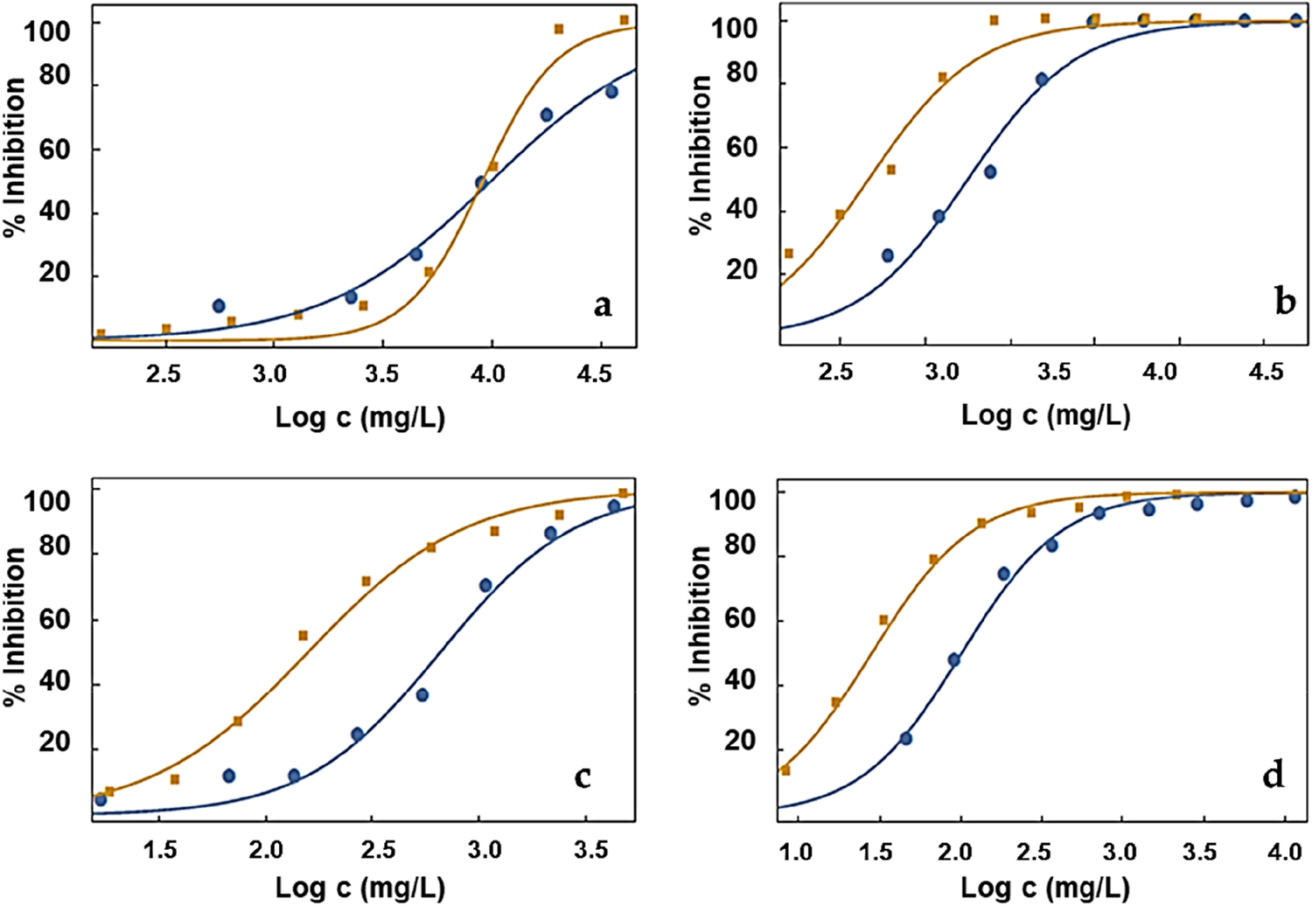
Comparison of the inhibition of bioluminescence of *A. fischeri* against the logarithm of the concentration of the four (blue circle) pure ionic liquids (**a**) EAN, (**b**) EIm NO_3_, (**c**) C_4_C_1_pyrr TFSI and (**d**) C_4_C_1_C_1_Im TFSI, and, after 30 min of exposure, with the corresponding (orange square) saturated lithium salt. Lines represent the fitting of the experimental results to Eq. (1)

The last part of this article is the analysis of the effect of the mixture of lithium salt and different ionic liquids. Figure 4 shows the comparison of the inhibition of bioluminescence of *A. fischeri* against the logarithm of concentration for 30 min of exposure of the four pure ionic liquids EAN, EIm NO_3_, C_4_C_1_pyrr TFSI and C_4_C_1_C_1_Im TFSI with the corresponding saturated lithium salt binary mixture, Li NO_3_ for the protic ILs and Li TFSI for the aprotic ones. The adjusted parameters of Eq. (1) and AAD values are shown in Table 8. Additionally, the values of EC_10_, EC_20_ and EC_50_ of these samples, pure ILs and mixtures, for 5 min, 15 min and 30 min of exposition are also shown in Tables 5, 6 and 7.

Changes in bioluminescence inhibition as a consequence of salt addition are strongly dependent on the ionic liquid–salt mixture in terms of EC_50_. Two behaviours can be observed, the first one for protic ILs, where the addition of lithium nitrate salt does not affect the toxicity or even reduce it, with regard to pure ILs and aprotic ILs, where the LiTFSI addition induces an increase in toxicity of mixture with regard to pure IL. Thus, pure EAN and EAN + LiNO_3_ mixture present results of EC_50_ at 30 min which suggests that the toxicity of the mixture is similar to that of the pure IL, although the inhibition of the lowest concentrations (EC_20_ and EC_10_) is lower for the mixture than for the pure IL. Likewise, the case of the mixture of EIm NO_3_ + Li salt is especially interesting. It presents lower toxicity than pure IL following the Passino and Smith classification since the EC_50_ at 30 min increases from 598 to 1073 mg/L for pure IL and mixture, respectively. This can be due to the different effects of IL and salt on the bacteria, being the salt relatively harmless (Viboud et al. 2012), reducing in the mixture the concentration of the most toxic part, the IL.

On the contrary, aprotic IL-lithium salt mixtures display a different behaviour than protic ones: The toxicity seems to increase significantly with the addition of lithium salt. Hence, the addition of Li TFSI reduced the EC_50_ of C_4_C_1_C_1_Im TFSI and C_4_C_1_Pyr TFSI by a factor of four in all the exposure times. As previously pointed out and taking into account that Viboud et al. (2012) classify the Li TFSI salt in the lowest toxicity group (EC_50_ > 1000 mg/L), this EC_50_ reduction with the lithium salt doping is unexpected, but can be explained by the augmentation of the concentration of TFSI^−^ ion after the addition of the salt to the IL with the common anion, which increases the hydrophobicity of the sample, closely related with the toxicity towards *A. Fischeri* bioluminescence, as pointed out previously in “Toxicity of pure ILs.” However, this important EC_50_ reduction is not enough to change the toxicity level after salt addition; slightly toxic and toxic for Passino and Smith and Chang criteria, as can be seen in Table 10.

**Table 10:** Toxicity identification of the IL-lithium salt mixtures using the criteria of Passino and Smith (1987) and Chang et al. (2013)

IL/mixture	Passino and Smith (1987)	Chang et al. (2013)
EAN	Relatively harmless	Non-toxic
EAN + LiNO_3_	Relatively harmless	Non-toxic
EIm NO_3_	Practically harmless	Non-toxic
EIm NO_3_ +LiNO_3_	Relatively harmless	Non-toxic
C_4_C_1_pyrr TFSI	Practically harmless	Non-toxic
C_4_C_1_pyrr TFSI + LiTFSI	Practically harmless	Non-toxic
C_4_C_1_C_1_Im TFSI	Slightly toxic	Toxic
C_4_C_1_C_1_Im TFSI + LiTFSI	Slightly toxic	Toxic

As a general observation, the addition of the lithium salts does not change the effect of these compounds on the bioluminescence of *A. Fischeri* bacteria, and the two criteria used in this study showed similar results although small differences can be found, especially due to the fact that Passino and Smith stabilised five levels of toxicity and Chang et al. only four.

Furthermore, it is important to point out that the extrapolation of these results to other toxicity endpoints cannot be done easily because the response is highly dependent on trophic levels (Perales et al. 2018). Whilst there are no references about ecotoxicity changes on ILs after salt addition, the results of Sixto et al. (2021) are of special interest. The aforementioned study analysed the effect of EAN and EAN-lithium nitrate mixture (equally harmless to *A. Fischeri*) on the respiration of two soils with different organic matter content, concluding that both pure IL and mixture strongly affect the soil respiration and that the organic matter content can mitigate the negative effect of lithium salt. All these observations highlight the need to increase and specify the knowledge of the impact of these ionic compounds and their mixtures in order to set up appropriate recovery and recycling procedures.

### Conclusions

In this work, the ecotoxicity of two protic ILs (ethylammonium nitrate and ethylimidazolium nitrate) and two aprotic ILs (butylmethylpyrrolidinium bis (trifluoromethylsulfonyl)imide and butyldimethylimidazolium bis (trifluoromethylsulfonyl)imide) pure and binary mixtures with different inorganic salts of electrochemical interest were tested towards changes on the bioluminescence of the bacteria *Aliivibrio fischeri*, using the Microtox® standard toxicity test.

The main conclusions of this work are as follows.

- Protic ILs are less toxic than aprotic ones, and that non-aromatic ones are generally less toxic than aromatic ones. Moreover, the role of water solubility is important, where lower toxicity is related to higher hydrophilicity in every group.
- Mixtures of EAN with the mono- and divalent salts do not significantly affect the toxicity of the mixture with regards to the pure ionic liquid, but the addition of an aluminium salt has an important effect on the bioluminescence of *A. Fischeri*, where this mixture is the most toxic of those studied.
- The effect of the addition of lithium salt strongly depends on the IL of the mixture: Protic ILs do not significantly modify the EC_50_ with regard to that of pure IL or even lead to slight increases of it, indicating a reduction of toxicity. Nevertheless, for the aprotic ILs, the effect of salt addition clearly increases the toxicity of the mixture in terms of EC_50_, although no changes on the toxicity step of the two criteria here applied can be observed.
- The two criteria used in this paper to classify the toxicity concerning marine bacteria *A. Fischeri* bioluminescence inhibition are comparable.

## Data Availability

Not applicable
